# Simulation and Evaluation of Cloud Storage Caching for Data Intensive Science

**DOI:** 10.1007/s41781-021-00076-w

**Published:** 2021-12-22

**Authors:** Tobias Wegner, Mario Lassnig, Peer Ueberholz, Christian Zeitnitz

**Affiliations:** 1 European Organization for Nuclear Research (CERN), Meyrin, Switzerland; 2 Hochschule Niederrhein, Krefeld, Germany; 3 University of Wuppertal, Wuppertal, Germany

**Keywords:** Cloud storage, Transfer simulation, Quality-of-service storage

## Abstract

A common task in scientific computing is the data reduction. This workflow extracts the most important information from large input data and stores it in smaller derived data objects. The derived data objects can then be used for further analysis. Typically, these workflows use distributed storage and computing resources. A straightforward setup of storage media would be low-cost tape storage and higher-cost disk storage. The large, infrequently accessed input data are stored on tape storage. The smaller, frequently accessed derived data is stored on disk storage. In a best-case scenario, the large input data is only accessed very infrequently and in a well-planned pattern. However, practice shows that often the data has to be processed continuously and unpredictably. This can significantly reduce tape storage performance. A common approach to counter this is storing copies of the large input data on disk storage. This contribution evaluates an approach that uses cloud storage resources to serve as a flexible cache or buffer, depending on the computational workflow. The proposed model is explored for the case of continuously processed data. For the evaluation, a simulation tool was developed, which can be used to analyse models related to storage and network resources. We show that using commercial cloud storage can reduce on-premises disk storage requirements, while maintaining an equal throughput of jobs. Moreover, the key metrics of the model are discussed, and an approach is described, which uses the simulation to assist with the decision process of using commercial cloud storage. The goal is to investigate approaches and propose new evaluation methods to overcome future data challenges.

## Introduction

Modern scientific experiments, such as ATLAS [1], CMS [2], Vera Rubin Observatory [3], or SKA [4], generate very large data samples. The large volume of these data samples typically requires distributed computing and storage resources [5–7] to process and store the data [8–10]. These resources are pooled in data centres and consist of different kinds of storage media with different Quality-of-Service (QoS) characteristics. Straightforward QoS deployments typically consist of disk and tape storage [11] targeting certain experiment needs, such as access latency, access pattern, or throughput. Additionally, constraints of the collaborating institutions, especially the costs, must be considered.

Tape storage typically comes with a high access latency but low cost per volume ratio compared to disk storage [12, 13]. A common use case of tape storage is archival and preservation of infrequently required data. The performance of tape storage is related to the effective throughput and depends on several factors, such as waiting time, mounting time, tape positioning time, data transfer duration, and unmounting time. The data placement strategy and the predictability of the data access pattern can significantly impact the overall performance of a tape system [11, 13]. Tape storage is the preferred storage type from a cost-oriented perspective, while disk storage is a preferred storage from a performance-oriented perspective.

In general, data intensive computing workflows require the use of performance-oriented storage, such as disks, because of the lower response time and better performance in concurrent and random access mode. This often results in maintaining at least one persistent copy of each input file on a disk system.

A typical workflow in scientific computing is the derivation of data to extract only the most important information and reduce the data volume, i.e., the volume of the input data of the derivation workflow is usually much larger than the volume of the output data. These workflows could be scheduled to be infrequently executed in bulk processing campaigns, e.g., whenever a new derivation software version is released. In this way, input files have to be read only once per campaign. Given this case, the input data would be preferably stored solely on tape. However, for larger collaborations, such as ATLAS, it is challenging to optimally organise those workflows into campaigns. For this reason, there are continuous derivation workflows that read input data more frequently.

To allow continuous derivation workflows to have an optimal throughput of input data, a common solution is to keep at least one copy of the vast majority of input files on a disk storage system. For example, almost all input data for the production of derivation data for ATLAS have one persistent copy on both tape and disk storage.

One approach that tries to take advantage of the infrequent usage of the input data for derivation production campaigns is implemented in the *data carousel* model [14]. The concept of the data carousel model is to transfer the input data from tape storage to disk storage, start processing the data, and continuously replace the data that has been processed by new data coming from tape.

Using this model, only a limited number of input files are required on disk storage at any one time. This allows removing the permanent copies of the input files from the disk storage system and storing the input files solely on tape storage. In this way, disk storage requirements are reduced to save cost or provide disk storage for other types of data.

The data carousel model was developed to improve storage usage and tape performance if the derivation workload is structured into campaigns so that the input files are required only once per campaign. With the continuous derivation workflow, the input files are accessed frequently, which would result in using the tape storage in concurrent random access mode, and thus significantly reduce the tape performance.

To reduce these limitations, the data carousel model can be combined with the *Hot/Cold Storage* model. The Hot/Cold Storage model categorises the storage in three different QoS categories: a large archival storage, a medium-sized cold storage, and small hot storage. The data is migrated between hot and cold storage based on a popularity metric. The concept is to use the cold storage as buffer for the archival storage to improve its performance, or as cache for the hot storage to reduce the number of re-transfers from the archival storage.

There are various ways to implement the cold storage, e.g., using on-premise storage, data lakes, or commercial cloud storage of different providers. Originally, the Hot/Cold Storage model was considered as an approach to integrate commercial cloud storage into the ATLAS infrastructure. For this reason, this contribution focuses on the implementation of cold storage by commercial cloud storage. Alternative options could be discussed in forthcoming publications.

In this contribution, we describe the *Hot/Cold Data Carousel* (HCDC) model, which is a combination of the Hot/Cold Storage model and the data carousel model. Furthermore, we present a simulation framework to evaluate the HCDC model. The HCDC model aims to minimise the disk storage required for derivation campaigns as achieved by the data carousel, while mitigating the negative impact on the tape storage performance for continuous derivation workflows.

Section 2 starts by defining the basic terminology. Subsequently, the main assumptions that were made for the evaluation are described. Finally, the Hot/Cold Storage and data carousel model are explained in more detail. Section 3 describes the HCDC model and lists possible variations of it. Section 4 starts by describing the architecture of the simulation software that was developed for the evaluation of the HCDC model. To validate the simulation, a simplified scenario was simulated and evaluated, and this is described following the architecture description. Finally, an explanation is given as to how the HCDC model was implemented in the simulation and which parameters were used. Section 5.4 shows the results of the simulation of the HCDC model and how the results were evaluated. We conclude in Sect. 6 with a summary and an outlook on future work.

## Fundamentals

Both the Hot/Cold Storage model and the data carousel model were developed based on the computing infrastructure of the ATLAS experiment, which obtains its resources from the *Worldwide LHC Computing Grid* (WLCG) [7, 15]. As mentioned before, globally distributed storage and computing resources are pooled in data centres. In the context of grid resources, those data centres are called *sites*. The storage resources of a site are logically grouped in *storage elements*. Storage elements could differ in the attributes of their underlying physical storage media or simply by the type of data they store.

As mentioned before, the Hot/Cold Storage model allows the usage of commercial cloud resources. The VR Observatory has already decided to use cloud resources from Google as an interim data facility in 2020/23 [16]. Moreover, ATLAS is investigating different approaches to adopt commercial cloud resources [17]. For this reason, the implementation and evaluation of the HCDC model have been performed considering resources from the *Google Cloud Platform* (GCP) [18]. *Google Cloud Storage* (GCS) denotes only the storage resources of the GCP.

Analogously to grid resources, clouds usually provide their resources pooled in *regions*, which represent the data centres. Storage resources are logically divided into *buckets*. In contrast to storage elements, cloud providers often allow buckets to be multi-regional. This means the data stored in a multi-regional bucket is transparently replicated across multiple data centres. In the scope of the Data Ocean project [17], the possibility of a scalable, globally accessible bucket was discussed with Google. Originally, the HCDC model was developed based on such a bucket. However, for the implementation presented here, the bucket is not required to be globally accessible. The possible options for implementing such a bucket still have to be investigated. A straightforward approach would be a transparent replication of the data in the bucket to regional data centres.

The HCDC model was evaluated based on the derivation workflow described in the introduction. The proposed model requires the workflows to be executed in one of two modes. The first mode assumes the derivation workflow is organised in predictable campaigns, resulting in an infrequent requirement of the input data. The other mode assumes the derivation runs continuously, which leads to a more frequent and less predictable demand for the input data. The existence of a popularity metric, such as the access frequency of a file, is assumed for the continuous mode.

### Data Carousel Model

The data carousel model aims at reducing the usage of performance-oriented storage like disk storage and prefers the usage of low-cost storage like tape storage. This is particularly applicable to workflows whose input data is required rather infrequently or for data with an easily-predicted access pattern. As is typically the case in scientific computing, the data carousel model requires that the workload can be divided into discrete units.

A derivation campaign starts with the definition of the workload. In ATLAS this is done by a production team creating *tasks* in the production system [19]. The production system coordinates with the data management system [10] and the workflow management system [20] the transfer of the data from tape storage to disk storage and the start of the data processing.

In data carousel mode, the input data is stored solely on tape. When the derivation campaign is defined and the data to process is determined, a *sliding window* is created. The sliding window has a specific size, e.g., the size of a given percentage of the total input data. The data that is required for processing must allocate space in the sliding window. After a successful allocation, the data can be transferred from tape to disk storage. When enough data has been transferred, the workload can start to process the data. The data is downloaded from the disk storage to the worker nodes, where it is processed by the derivation software. When the processing of the data completes, the corresponding data is deleted from the disk storage and de-allocated from the sliding window. Using this approach, only disk storage equal to the sliding window size is required at any one time.

The possible size of the sliding window is limited by the following parameters:

- available storage for the sliding window
- volume of input data to process
- throughput and latency of the tape storage
- time between start of transfers and start of workloads
- available computing resources

The minimal and maximal size of the sliding window depends on the available storage and the volume of the required input data. Typically, the volume of the input data is larger than the storage available for the sliding window. Thus, the temporary storage available for processing is the limit rather than the volume of the input data. The window size must be large enough to hold all the input data for all currently running jobs.

Another potential limitation of the size of the sliding window is given by the performance from the tape storage to the disk storage and the time it takes to process the data. For example, if the performance from the tape to the disk storage is the bottleneck, a very small sliding window size would be sufficient. The reason is that a large window could not be filled up.

### Hot/Cold Storage Model

**Fig. 1:**
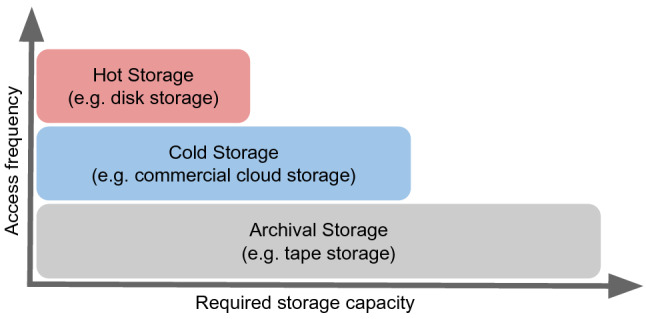
Hot/Cold storage model. One replica of each file is stored on archival storage. Files are migrated between cold and hot storage based on a popularity metric

As shown in Fig. 1, the Hot/Cold Storage model divides the storage into hot storage, cold storage, and archival storage. The main dimensions in which the requirements to the storage categories differ are the storage capacity and a popularity metric, such as the access frequency of the data estimated by the number of times a file has been used.

Hot storage requires a small capacity to store only the most frequently accessed data. Optimally, the hot storage would be located in close geographical distance to the computing resources to allow a high bandwidth and low access latency connection. Regarding the QoS properties, the storage implementing the hot storage must provide good performance in terms of throughput and access latency, especially in concurrent and random access mode.

Cold storage requires a larger storage capacity than hot storage. There are two use cases for cold storage. First, it can be used as a temporary buffer when the hot storage is full. In this case, cold storage accepts data from archival storage that is required or is likely to be required based on the popularity metric. Second, it can be used as a cache between the archival and the hot storage. In this case, the cold storage caches the data from the hot storage that is no longer required on the hot storage but is likely to be required again in the short term.

Archival storage requires the largest capacity. The QoS properties of archival storage typically describe a higher access latency and significantly lower performance for concurrent and random access mode. The Hot/Cold Storage model assumes that at least one replica of each file is kept in an archival storage.

Various approaches are possible to use the different storage categories together. The data on hot storage is replaced very frequently. The data is preferably transferred from a cold to a hot storage. If the required data is not available on a cold storage, it is transferred from an archival storage.

In the implemented variation of the Hot/Cold Storage model, the required data that is not available on the cold storage is directly transferred from the archival to the hot storage. Prior to the deletion of data from the hot storage, the data is replicated to the cold storage. Using this approach, cold storage is only allocated when it is really required, which can result in saving storage cost. Alternatively, the required data could firstly be transferred from the archival to the cold storage and then be transferred to the hot storage. This would result in less delay for the deletion because the data does not have to be transferred to the cold storage first. However, it would increase the waiting time for the required data.

Another point is how the allocation and deallocation of cold storage are managed. Ensuring the existence of hot storage data on cold storage prior to its deletion requires either an unlimited cold storage capacity or a deletion strategy of cold storage data. Another approach would be to set a threshold based on the popularity metric and only transfer data to the cold storage that has a certain popularity. This threshold could be used to improve the hit/miss ratio when using the cold storage as cache. The implemented version of the model assumes an unlimited cold storage.

## HCDC Model

The HCDC model joins the Hot/Cold Storage model with the data carousel model. The model includes two types of resource providers. First, the institution or company using the model. Resources of this provider are persistently available and can be used independently of the cost, e.g., the institutions that are associated with the ATLAS experiment provide a pledged amount of resources.

Second, commercial cloud providers providing resources with typical cloud behaviour, i.e., they can be allocated and deallocated on demand to an arbitrary extent. However, they introduce different kinds of costs. Regarding storage resources, there are at least costs for the stored volume per time, depending on the storage type. Furthermore, costs for operations like writing, reading, deleting, or changing metadata of files, are typically charged. Another cost factor is the network traffic. Usually, cloud providers only charge for egress and traffic between different regions within the cloud. The amount charged for traffic within the same cloud depends on the source and destination endpoints. Egress traffic out of the cloud to the internet is usually the most expensive.

The Hot/Cold Storage model and the data carousel model can be combined in different variations. The first consideration is whether the derivation workload operates in continuous mode or is executed in campaigns. For organised campaigns, the data carousel part is expected to be most effective, whereas the Hot/Cold Storage part should be less important. As described in Sect. 2, this is because for organised campaigns the data is required infrequently and the order to read the files can be well planned before starting the workload. In this case, the potential benefit from the Hot/Cold Storage model part would be to use the cold storage as a prefetching area. This could avoid the performance of the archival storage becoming a bottleneck after the start of the campaign. In continuous production mode, the data is required more frequently and less predictably. This leads to the expectation that the data carousel part is less important. However, the Hot/Cold Storage part should become more important in providing a cache for the processed data and reduce the data access from archival storage.

Another point to consider is which storage category of the Hot/Cold Storage model is represented by the cloud storage. This depends on the different storage types offered by the cloud provider and should be decided based on the QoS properties described in Sect. 2.2.

Using the cloud storage as an archive would necessitate the acquisition of the largest amount of cloud storage among all three storage categories. However, some cloud providers offer different storage types with different pricing policies. For example, GCS offers storage types with a low cost per volume ratio, but a higher cost per access ratio. Since the archival storage category is used for the least frequently accessed data, the higher cost per access ratio could be negligible and the lower cost per volume ratio could be beneficial.

Using the cloud storage as hot storage would reduce the required cloud storage volume. Since hot storage requires high-performance storage, the cost per volume for the storage type would typically be higher. Furthermore, the egress cost would be very high if the data is processed outside of the cloud because the hot storage contains the most popular data.

Cold storage has a more flexible volume requirement, depending on the popularity metric and the available hot storage. With cloud storage as cold storage, the egress cost would depend on the number of reusages of the data and the available amount of hot storage.

Using the cloud for cold storage enables the sliding window of the data carousel model to become dynamic. That means that, in case hot storage is full, the sliding window can be extended by using cold storage to keep an optimal performance of the archival storage.

**Fig. 2:**
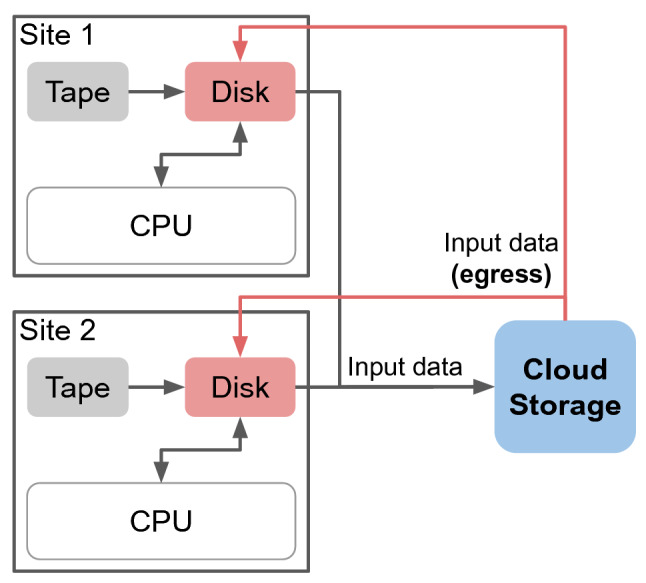
Schematic illustration of the combination of the Hot/Cold Storage model and the data carousel model with two sites

Figure 2 illustrates how the Hot/Cold Storage model could be combined with the data carousel model, for the simple case of two sites. The storage categories of the Hot/Cold Storage model part are assigned straightforwardly using site tape storage as archival storage, cloud as cold storage, and site disk storage as hot storage.

To read data from a tape system on the grid, the data always has to be transferred to a small disk storage buffer area first. The graphic shows the disk storage summarised in a single box per site. In this model, the data on tape is read only and there is no data added to tape.

The CPU box represents the computing nodes with their local storage areas. The compute nodes receive their input data only from the disk storage of the associated site. The output data is uploaded to a disk storage area of the site.

Input data that has been processed or does not fit on the hot storage is transferred to the cloud storage. The ingress is assumed to be free of charge, while the egress from the cloud storage to the disk storage is charged. The deletion of the data at the cloud storage can be implemented either with an expiration time or based on a storage limit and the popularity metric.

## Simulation Framework

A simulation was developed to evaluate an implementation of the HCDC model. In addition, the simulation can be used to analyse different models and scenarios combining grid and commercial cloud resources. Its main feature is the modelling of storage and network resources by simulating transfers.

The simulation is based on events that are scheduled to discrete time points. An event is a subprogram that is executed at its scheduled time point during simulation runtime. An internal clock produces the discrete time points. The smallest time step the simulation can operate on is one second.

After an initialisation phase, the simulation runs an event loop. Each repetition of the event loop handles all events of one time point. Thus, every iteration of the event loop increases the simulation clock by the difference between the scheduling time of the events of the current and next iteration.

### Architecture

A first prototype of the simulation was implemented using Python. In favour of controlling the memory management and the performance when iterating data, the simulation was finally developed in C++. Configuration files are formatted in JSON.

**Fig. 3:**
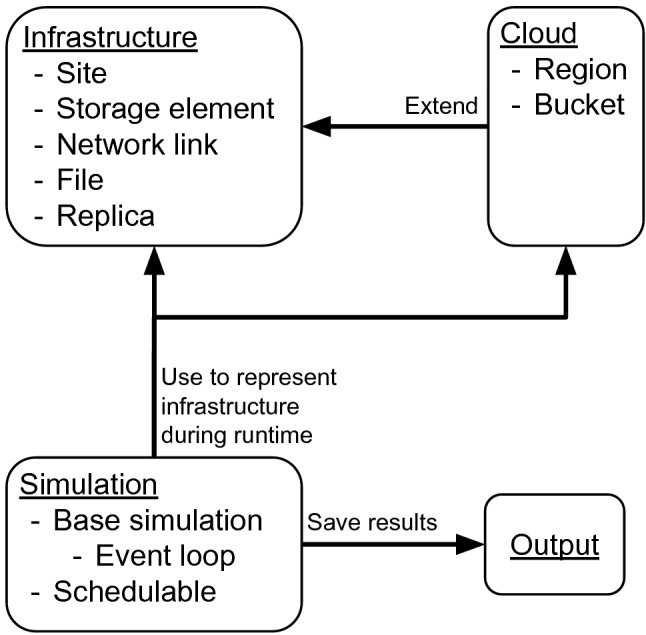
The four modules of the simulation. Simulation is the central module that manages the control flow and uses the other modules to create, manage, and save the simulation data

The simulation consists of different modules which can be divided into four topics as shown in Fig. 3.

- The infrastructure module provides classes to represent the infrastructure that is simulated, e.g., storage elements, network links, files, or replicas.
- The cloud module extends classes of the infrastructure module to provide functionality to simulate commercial cloud resources.
- The simulation module provides classes that execute and control the simulation flow.
- The output module persistently stores the data generated by the simulation.

The primary classes of the simulation module are the BaseSimulation and the Schedulable class. Both of the two classes are designed to be specialised in derived subclasses. The specialisation of the BaseSimulation-class defines the initialisation and execution of the simulation, including the execution of events and the management of the simulation clock. The Schedulable-class serves as the base class for every event that needs to be scheduled during the simulation run. The initial events are scheduled by the subclass instance of the BaseSimulation-class. When events are executed, they can reschedule themselves or create and schedule new events depending on their implementation.

The infrastructure module contains data structures for all entities required to set up the infrastructure of the model. Storage elements are objects that address a storage area and describe its properties. They also store run time data of the simulation, e.g., volume used and stored replicas. Each storage element is associated to one site. Sites can contain different storage elements. Network links represent the connection between storage elements. During run time, they store a reference to the source and destination storage element, as well as the traffic introduced by transfers and the number of currently active transfers. Files describe the data that can be transferred, e.g., a file object stores the file size and the expiration time. The expiration time is the time at which a file gets deleted. Replicas represent stored data. During run time, a replica object contains a reference to a storage element and a file, which means that the file is stored at the storage element.

Basic functionality is covered by built-in implementations in the simulation module and can be used by customising configuration files. For example, the HCDC model does not need a special implementation of the BaseSimulation-class because the built-in implementation can set up the required models based on corresponding configuration files.

A common approach to implement a new model for the simulation is using two configurable types of events. One type is called *transfer generator* and defines the logic of how transfers are generated. The other type is called *transfer manager* and is used to update and keep track of the states of the generated transfers. Based on their implementation, these events respectively create new transfers and update existing transfers. The events reschedule themselves using a configurable time interval.

For all models with a transfer simulation based on bandwidth, throughput, or transfer duration, it should be sufficient to define the parameters in a configuration file and use one of the built-in transfer manager implementations. The transfer generator implements much more logic and thus needs a specialised implementation for each different model.

There are two built-in implementations available for the transfer manager. The first one increases the size of the destination replica of each transfer. The amount by which the size of the destination replica is increased is calculated based on the time since the last update and on the configured throughput or bandwidth of the network link.

A network link can be configured in one of two modes. A configuration with a bandwidth will equally divide the available bandwidth among the number of active transfers. A configured throughput is not divided and is independent of the number of active transfers. Thus, a throughput requires a reasonable distribution of the number of transfers to deliver realistic results.

The other built-in transfer manager implementation updates the destination replica based on a configured transfer duration, i.e., the transfer manager increases the destination replica by a fixed increment each tick to ensure the replica is complete after the configured transfer duration.

### Validation

To validate the basic functionality of the simulation, the transfers of the ATLAS derivation input data were simulated. Since this is a process that is already running in production, there is sufficient data available to calculate parameters for the simulation and to provide a scale for the output.

The validation scenario was implemented in the simulation by creating a new transfer generator. This transfer generator is built to only create transfers from a configurable set of source storage elements to a configurable set of destination storage elements. In each tick, the transfer generator processes three steps. First, it determines the number of transfers to generate for each source/destination pair. Second, it uniformly randomly selects source files that do not already exist at the destination. Finally, it creates the corresponding number of transfers using the selected source and destination information.

**Table 1:** Parameters and their configuration for the simulation validation scenario

Parameter	Value/Configuration
Simulated time	59 days 19 hours
Transfer mgr. update interval	1 s
Transfer gen. update interval	10 s
No. sites	3
No. initial replicas	1000 per site
No. network links	2 per site
Throughput	8.10 MB/s per network link
File size	Exponentially distributed:
	$$\lambda = 0.61972$$
	$$10.23 \text { MB} \le \text {size} \le 13.73 \text { GB}$$
No. transfers generated	Exponentially distributed:
	$$\lambda = 3.33437$$

Table 1 lists all parameters and their configuration that were used to simulate this scenario. The data to calculate values for these parameters was taken from the ATLAS distributed computing monitoring system. This monitoring system only provides the data of the past two months. For this reason, the simulated time frame was set to almost two months, as shown in the table. Specifically, the transfer data from 2020-05-30 10:00:00 to 2020-07-29 05:00:00^1^ of the three sites with the highest number of transfers during this period was collected.

Five data samples of the monitoring data were considered for the validation. A sample of the file size, a sample of the number of transfers, and a sample of the transfer throughput were used to calculate the parameters for the model. The samples of the transferred volume and transfer duration were used to validate the output of the model.

The monitoring system provided the data of all five samples only in an aggregated form. For the file size distribution, the data was aggregated in the form of a histogram. This histogram provided the number of files per file size. To obtain the best data resolution, the smallest possible histogram bin width of 128 MB was used. For the other metrics, data was aggregated in date time histograms with the smallest possible bin width of 1 hour.

The simulation parameters for the file size distribution and the number of transfers were calculated by fitting a random distribution function to the data. The fitting was done by maximising a log-likelihood function. Different distributions were considered. The exponential distribution showed the best fit for both metrics. This can be attributed to the nature of the distribution providing a moderate average with occasional low and high values. This property describes well both the file size distribution and the number of transfers.

The throughput parameter was calculated by using the overall throughput from the date time histogram of the 3 observed sites. To calculate a throughput value, the mean of the histogram was taken and equally distributed by dividing it by 3.

As mentioned, the monitoring data for the number of transfers was available in a date time histogram with 1 hour wide bins. In reality, the transfers are not only created every hour but distributed across this hour. For this reason, the data was linearly interpolated to satisfy the 10 seconds update interval of the transfer generator. Furthermore, the data was uniformly distributed across the 6 network links because the data from the monitoring system was aggregated. The interpolated data was used for the fit to the random distribution functions.

The transfer generator and transfer manager update intervals control how frequently new transfers are created, and existing transfers are updated. These parameters can influence the resolution of the simulation. Thus, finding the optimal value means finding a trade-off between simulation run time and simulation precision.

The time complexity for a single update of the transfer manager scales linearly with the number of active transfers of all network links. From the monitoring data, it was known that the number of transfers per hour is on the order of 1000. The largest bin of the transfer duration histogram from the monitoring data reached $\approx 10$ minutes. Given these values, it is not expected that the number of active transfers reaches a scale which would noticeably increase the simulation run time. This allowed setting the transfer manager update interval to the lowest value of 1 second to achieve the highest resolution.

The transfer generator update interval defines the minimum difference between the creation time of transfers that were not created at the exact same time. Choosing an excessively large update interval would result in a high but infrequent number of transfer generations. Conversely, a too small update interval could result in an increase of the simulation run time. An update interval smaller than the time between two transfer generations does not improve the resolution. Thus, a minimum update interval of 1 second is not reasonable for an expected rate of 1000 transfers per hour. With a value of 10 seconds, the overall simulation run time was on the order of $\approx 30$ seconds. The mean of the distribution function of the number of transfers to generate is $1/\lambda = 1/3.33437 \approx 0.3$. This means that on average, using the 10 seconds interval a new transfer is generated every third update.

At the start of the simulation, each storage element is initiated with 1000 replicas. The transfer generator randomly selects a replica as source for each transfer. Only files that do not have a replica at the destination and are not in the process of being transferred to the destination can be selected. After a completed transfer, the destination replica is deleted again to allow transferring the replica again. The 1000 replicas provide a sufficient pool of selectable replicas. In case no replica meets the select conditions, a new replica is created.

**Table 2:** Results of simulation correctness validation. The RWD column shows the real-world data. The Sim column shows the simulated data

Metric	RWD	Sim	Unit	Diff. (%)
File size	1.74	1.73	GB	0.57
No. transfers	1.77	1.80	No./10s	1.69
Throughput	8.10	8.01	MB/s	1.11
Traffic	3.01	3.11	GB/s	3.32
Transfer duration	212.18	214.10	s	0.90

The evaluation was made by using the parameters from the distributions fitted to the real-world data as input and comparing the average of the output parameters. It was not necessary to run numerous iterations of the simulation. Five runs have been executed, and the output was compared to verify this. The standard deviation of each observed metric was between 0% and 0.07%. The standard error for the different metrics did not exceed 0.03%.

Table 2 shows the observed metrics with real-world data values and the values simulated, as well as the differences between them. As explained before, the values simulated are the mean of five different simulation runs. The file size in gigabytes is the mean value of the file sizes. The number of transfers is the number of transfers that were finished every 10 seconds. The throughput is the mean value of the throughput of all transfers equally distributed to the three sites. These parameters are taken from the real-world data and are used as input parameters for the simulation. Based on these parameters, the traffic and transfer durations are computed during the simulation and are observed as output metrics. The traffic is the summed data volume that is transferred with each transfer manager update. Thus, the throughput metric is calculated per transfer, while the traffic is calculated time based. The transfer duration is the mean duration of each transfer. The greatest difference between real-world data and simulated data is the traffic metric with 3.32%.

## HCDC Simulation

The HCDC model features several more cases and conditions to generate transfers than the straightforward random selection of files from the model of the previous section. The following sections explain the implementation of the HCDC model in the simulation software, the parameters used, and the evaluation of the results.

### Site Configuration

**Fig. 4:**
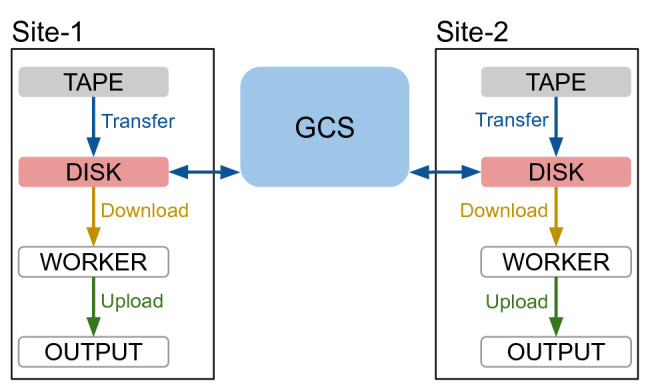
Implementation of the HCDC model in the simulation. It shows the configured storage elements for each of both sites and the network link setup. The network link labels name the type of transfer. GCS illustrates a single cloud bucket used by both sites

Figure 4 shows the HCDC model implementation for the simulation. It is based on the two grid sites, Site-1 and Site-2, each with four different storage elements. Network links in the simulation are always directional. The arrows illustrate the network links and their directions.

The GCS box represents a GCS bucket. In the simulated scenarios, each site uses only the data originating from its own tape storage element, although both sites have access to all the data on GCS. To enable sites processing data from other sites, a study would be required of how to split the workload exactly. A possible approach would be to use a given share, e.g., $80\%$ of the jobs use local site data and $20\%$ of the jobs use remote site data. Another approach could use the popularity metric to additionally select remote site data.

The simulation was configured with the following storage element types.

**TAPE** storage elements provide tape storage that represents the archival storage of the Hot/Cold Storage model. The tape storage contains one replica of each file, and thus represents the origin of all input files. Typically, requests to tape are kept in a queue for some time if the tape is not mounted, to optimise the reading requests. Additionally, there is a latency for mounting, positioning, and dismounting the tape. These delays are simulated by configuring the tape storage elements with an access latency. This means when a queued transfer from tape storage becomes active, the start of the actual data transfer is deferred based on the access latency.

**DISK** storage elements provide disk storage exclusively for input data and represent the hot storage of the Hot/Cold Storage model. The disk storage is used as source for the downloads of the derivation production input files to the worker nodes. The disk storage represents the storage with the best connection to the worker nodes and provides high bandwidth and low latency access. The disk storage serves as cache layer for the worker nodes. For this reason, the worker nodes download their input files only from the disk storage element in the simulated scenario. The disk storage represents the storage area of the sliding window in terms of the data carousel model.

**WORKER** storage elements are used to simulate the local storage of the worker nodes. The simulation was designed to represent transfers with storage and network resources, so there is no default functionality for computing resources. It is assumed that the input files always fit entirely on the worker node. Thus, worker storage elements must not have a limit set. In general the HCDC model can also be used without this assumption, but the simulation implementation would require several adjustments, e.g., to support streamed data input.

**OUTPUT** storage elements that represent a storage area exclusively for output data. Output storage elements are used to store the output files of the jobs which will be uploaded from the worker node storage.

**GCS** buckets are a special type of storage element used to represent the cold storage of the Hot/Cold Storage model. The cloud module of the simulation contains a GCS implementation. This implementation allows creating GCS buckets. GCS buckets are storage elements that are extended by certain functionalities like storage increase/decrease tracking, ingress/egress tracking, and cost calculation. These functionalities reflect the cost model of the cloud provider.

There are two special transfer types in this model. First, the transfers from disk storage elements to worker storage elements are called *download* instead of transfer. Second, the transfers from the worker storage element to the output storage element are called *upload* instead of transfer. The difference between a download and a transfer is that the downloaded replica is not managed by the data management system anymore. Correspondingly, an upload creates a new managed replica in the data management system. Furthermore, in the simulation downloads and uploads are not processed by the transfer manager, and they are stored in a different format in the output module.

### Simulation Workflow

As explained in Sect. 4.1, a transfer generator implements the main part of a model in the simulation. The HCDC transfer generator is implemented and configured to simulate continuous derivation production. The transfer generator simulates the submission and execution of jobs. A job has a selected input file, which is transferred from tape to disk, downloaded to the worker storage, processed, and subsequently deleted from the disk storage.

**Fig. 5:**
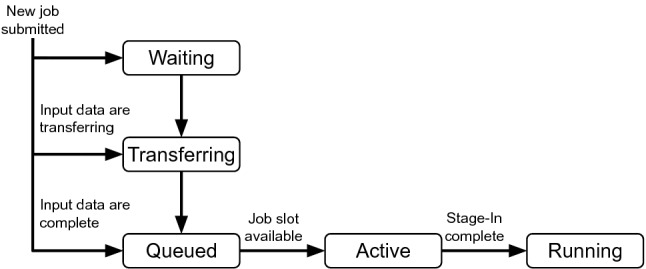
State transitioning of jobs during production phase

The transfer generator implements the derivation workflow by using *job objects* that transit through a state machine. Figure 5 illustrates the state machine. Each job object is in one of the states waiting, transferring, queued, active, or running. The arrows indicate the transition between the states. The arrow labels are the conditions for a state transition.

The first operation of each transfer generator update is the submission of new jobs. A number of new jobs, which is based on the configuration, are submitted. For each new job, the input data is randomly selected based on the popularity. In the following, the various states and transitions are explained in more detail.

**Waiting:** The required input data is not at the disk storage element. No transfer for the input data to the disk storage element exists. Not enough disk storage is available to create a transfer of the required input data to the disk storage. When disk storage becomes available, a transfer is queued and all job objects that are waiting for these data enter the transferring state. Job objects in the waiting state are processed in first in, first out order.

**Transferring:** The required input data is not completely available at the disk storage element, but a transfer is queued or running to replicate the data to the disk storage element. When the transfer is complete, the state of the job object changes to queued.

**Queued:** When the input data of a job is already at the disk storage element or a transfer for the input data is completed, the job object state transits to the queued state. In this state, the job waits for compute resources in the form of job slots to become available. The simulation can be configured to provide a specific number of job slots per site. If a job slot is available, the job object state changes to active.

**Active:** In this state, a job is occupying a job slot. A download of the input data from the disk storage element to the worker storage element is started. When the download is finished, the details about the download and the job object are stored in the simulation output and the job enters the running state.

**Running:** The job object simulates the derivation job execution. Job objects in the running state are not regularly updated anymore. They are paused for a randomly generated job execution duration, during which time the job object is waiting for the job to finish. Subsequently, uploads of the output data are created, and the job object is deleted.

The deletion of data that is no longer required is processed at the beginning of each transfer generator update. Obsolete replicas on the disk storage element that already have a replica on the GCS bucket are deleted immediately. The data that is not available on the GCS bucket is migrated there, i.e., transferred from the disk storage element to the GCS bucket and subsequently deleted on the disk storage element.

### Parameters

**Table 3:** Parameters and their configuration for the simulation of the HCDC model

Parameter	Value/Configuration
Simulated time	90 days
Transfer mgr. update interval	1 s
Transfer gen. update interval	10 s
No. sites	2
No. initial replicas	$10^6$ per site
Popularity	Geometrically distributed:
	$$p = 0.1$$
	$$1 \le x < 50$$
Input file size	Exponentially distributed:
	$$\lambda = 0.026$$
	$$9.76 \text { MB} \le \text {size} \le 134 \text { GB}$$
No. jobs submitted	Normally distributed:
	$$\mu = 0.63366$$
	$$\sigma = 0.37292$$
	$$n \ge 0$$
Job duration	Exponentially distributed:
	$$\lambda = 0.00409$$
	$t \ge 16.666$ minutes

Table 3 shows the general and site specific parameter values used for the HCDC model. The monitoring data was taken from different sources for the HCDC model. Data to calculate the throughput was taken from the transfer monitoring data. The other parameters such as input file size distribution were taken from the job monitoring data. Contrary to the transfer monitoring data, the job monitoring data is not limited to the past two months. However, since the mean throughput is calculated from the data, it is assumed that the mean value is also representative for the time of three months. The monitoring data for two months was taken for the time from 2020-07-08 12:00:00 to 2020-09-06 12:00:00. The monitoring data for three months was taken for the time from 2020-06-08 12:00:00 to 2020-09-06 12:00:00.

First real-world tests with the HCDC model would be limited to rather small scale deployments, especially in terms of the number of sites. This prevents a large waste of resources in case of issues with the implementation. Additionally, it allows acquiring an impression of the model without incurring excessively large cloud cost. The monitoring data showed that in total 80 sites processed $\approx 6.5$ million derivation jobs during the observed 3 months. The 2 sites with the most number of derivation jobs each processed $\approx 0.5$ million jobs. To be able to use the same job submission configuration and to keep the first evaluation of the model clear, only these 2 sites were simulated. The simulated time was set to 90 days according to the monitoring data.

The required real time to simulate one day was typically below one second. Thus, a value of 1 second could be used for the transfer manager update interval to achieve the best possible resolution. The vast majority of the simulation run time is spent in the transfer generator. The transfer generator update interval was chosen for the same reason as in Sect. 4.2 but based on the number of submitted jobs.

The file size distribution was calculated using the same approach as in Sect. 4.2. The monitoring data used did not provide the size of each single input file but only the total input volume of each job. Using this monitoring data makes the assumption that one transfer from the tape storage provides data for at least one job, i.e., for each job the tape access latency and overhead is considered at most once. The monitoring data showed that 50% of the jobs downloaded fewer than 5 files and 80% fewer than 15 files. However, this assumption implies a reasonable data placement on tape and that the workflow management system is able to structure jobs to transfer data bunches from tape and process them. These implications are still challenging objectives. To improve their impact on the accuracy of the simulation, a more detailed model of the job submission would be required. Furthermore, a more detailed tape model including the data placement strategies used would be required.

Since one file corresponds to the input data of one job, the number of initial replicas can be based on the number of jobs. The number of finished jobs in the monitoring system was $\approx 10^6$. Thus, an equal number of files were created.

As mentioned before, the number of times data was processed is used as the popularity metric. This information can be collected from the central production database. Most data was processed once, exponentially falling-off to 50 times processed. A geometrically distributed random function can approximate this falloff with the chosen success probability parameter of $p = 0.1$ and a limitation of the values between 1 and 50.

The number of jobs to submit is generated by a normally distributed random function. The duration of each job is generated by an exponentially distributed random function. The estimation of the parameters of these functions follows the same approach as the number of transfer generation in Sect. 4.2. Since the number of jobs to submit is fitted to the monitoring data, no job slot limitation is configured.

No specific configuration for the number of output files and volume of the output data was used. This is because in this model, these metrics do not affect the bandwidth or cost. Only the job slot is blocked until all uploads of the output files are finished. Furthermore, the metrics depend directly on the number of jobs finished, and thus could be calculated after the simulation.

**Table 4:** Network configuration of the simulated model

Site	Source	Dest.	Max. active	Value (MB/s)
Both	GCS	Disk	100	294.00
Both	Disk	GCS	100	500.00
Both	Disk	Worker	N/A	88.24
Site-1	Tape	Disk	100	22.62
Site-2	Tape	Disk	100	62.35

The other parameters define the network configuration. Table 4 shows the values used. The first two rows show the throughput for the network links between the GCS bucket and the disk storage elements. The third row shows the throughput for downloads. These links are configured equally for Site-1 and Site-2. For transfers managed by the transfer service, the maximum number of active transfers was limited to 100 according to the real world limits. The number of downloads is not explicitly limited.

For the throughput parameters, the mean value of the monitoring data was taken. Compared to the transfers between the disk storage element and the GCS bucket, the download throughput seems to be low, but there is no limitation on the number of active downloads. As explained in Sect. 4.1, values configured as throughput are not divided among the number of active transfers.

For the throughput from and to the GCS bucket, only monitoring data from small scale manual tests was available. Because of the small scale, these values might contain uncertainties and must be adjusted when larger scale data is available.

Another configuration parameter to consider is the access latency of the tape storage elements. Creating a proper model to estimate the access latency would be a complex topic itself and would require detailed log data from real tape systems. For this reason, a constant average value of 30 minutes was used for the access latency. In addition, tests were done using normally distributed random values for each transfer.

The pricing information of the commercial cloud storage is defined in a configuration file. It contains the pricing details of different storage categories, different grades of cost depending on the volume stored, and the network cost depending on the egress destination. The file was created based on the public pricing data from the GCP documentation on 2020-09-10. For the simulation, the standard storage class for a regional bucket was used.

ATLAS and the VR Observatory have worked on research and development projects in collaboration with Google. During this work, special prices were negotiated. Furthermore, Google supports different peering methods than using the internet. Network cost can be reduced by connection to GCP using a non-public network. For example, the price for downloading to the internet in Europe is between 0.08 and 0.12 USD/GiB. By using the direct peering option, the cost is reduced to 0.05 USD/GiB in Europe. The interconnect peering option charges only 0.02 USD/GiB [18]. These peering methods typically require a physical network connection to an internet exchange point. Although interconnect peering might reduce the GCS network cost, this could also introduce new costs depending on the contracts with the providers of the existing network infrastructure.

**Table 5:** Different storage limits per configuration

Cfg.	Disk limit	GCS limit	Tape limit
*I*	N/A	0	N/A
*II*	100 TB	0	N/A
*III*	100 TB	N/A	N/A

Three different configurations of the HCDC model were simulated. All configurations use the same job submission, file, and network parameters. Since these parameters are fitted to real-world data, the limits are known to be achievable by the real-world system. The differences between the configurations are the storage limits of the disk storage elements and the GCS bucket. Table 5 shows the storage limits per configuration.

***Configuration I*** has the limit of the GCS bucket set to zero to prevent the usage of the GCS bucket. In addition, no limit is set on the disk storage elements. With this configuration, all input data is transferred from the tape storage element to the disk storage element and is kept at the disk storage element. The simulation results should be comparable to the current ATLAS derivation production workflow since the only difference is the initial transfer from the tape storage element to the disk storage element.

***Configuration II*** has the same limit value of zero on the GCS bucket. In addition, a limit of 100 TB is set on each disk storage element. This configuration of the model shows how the results would be if there was no cloud storage to cache the input data. In this scenario, the input data must be transferred from the tape storage element to the disk storage each time the data is required.

***Configuration III*** is without a limit on the GCS bucket, but with the limit of 100 TB on the disk storage elements. In this way, the fully combined model is simulated with all storage areas usable. This configuration allows analysing the difference when adding the cloud storage as cache.

### Evaluation

The first metric that was evaluated is the number of finished jobs. From the data of the monitoring system, it is known that the number of finished jobs should be $\approx 10^6$. As explained in Sect. 5.3, it is expected that the results of *configuration I* are similar to the real-world data. In *configuration II*, the data on the disk storage element is deleted after it has been processed because of the limit of disk storage. In addition, the GCS bucket is not used. This leads to transferring the data from the tape storage element to the disk storage element each time the data is required. Compared to *configuration I* this results in an increase of total required transfers. With the increased number of transfers and the additional access latency for tape access, *configuration II* is expected to show a decrease in the number of finished jobs.

**Table 6:** Mean number of finished jobs and mean volume downloaded with their standard deviation for *configuration I, II,* and *III* of 20 simulation runs. The number in the brackets is the corresponding standard error (SE)

Cfg.	No. jobs done (SE)	Download volume (SE)
*I*	996k ± 0.05% (0.01%)	41.11 PB ± 0.20% (0.04%)
*II*	853k ± 0.11% (0.02%)	35.28 PB ± 0.24% (0.05%)
*III*	996k ± 0.05% (0.01%)	41.02 PB ± 0.38% (0.08%)

Table 6 shows the simulated number of finished jobs, the total downloaded volume, and the corresponding standard error (SE) for *configuration I, II,* and *III*. Comparing the results of *configuration I* and *II* already gives an impression of the expected impact of the limit on disk storage. The limit results in $\approx 15\%$ fewer jobs finished and in $\approx 14\%$ less input volume downloaded.

It is conceivable that the difference between the number of finished jobs of *configuration I* and of *configuration II* increases as the simulated time frame increases. The explanation is that in the beginning both configurations behave similarly because all the data is on tape storage. At some point in *configuration I*, all the data will be available on disk storage. Thus, the data can be directly downloaded from the disk storage without transferring it from tape first. This is not the case for *configuration II* because of the disk storage limit. With a limited disk storage, further jobs can only be started if sufficient disk storage space is made available by the running jobs.

*Configuration III* uses the same values as *configuration II* except that the GCS bucket has no limit. As Table 6 shows, the results are almost equal to *configuration I* in terms of number of jobs finished and volume downloaded. That means, in terms of these metrics, the cloud storage is able to compensate for the limit of the disk storage element.

**Fig. 6:**
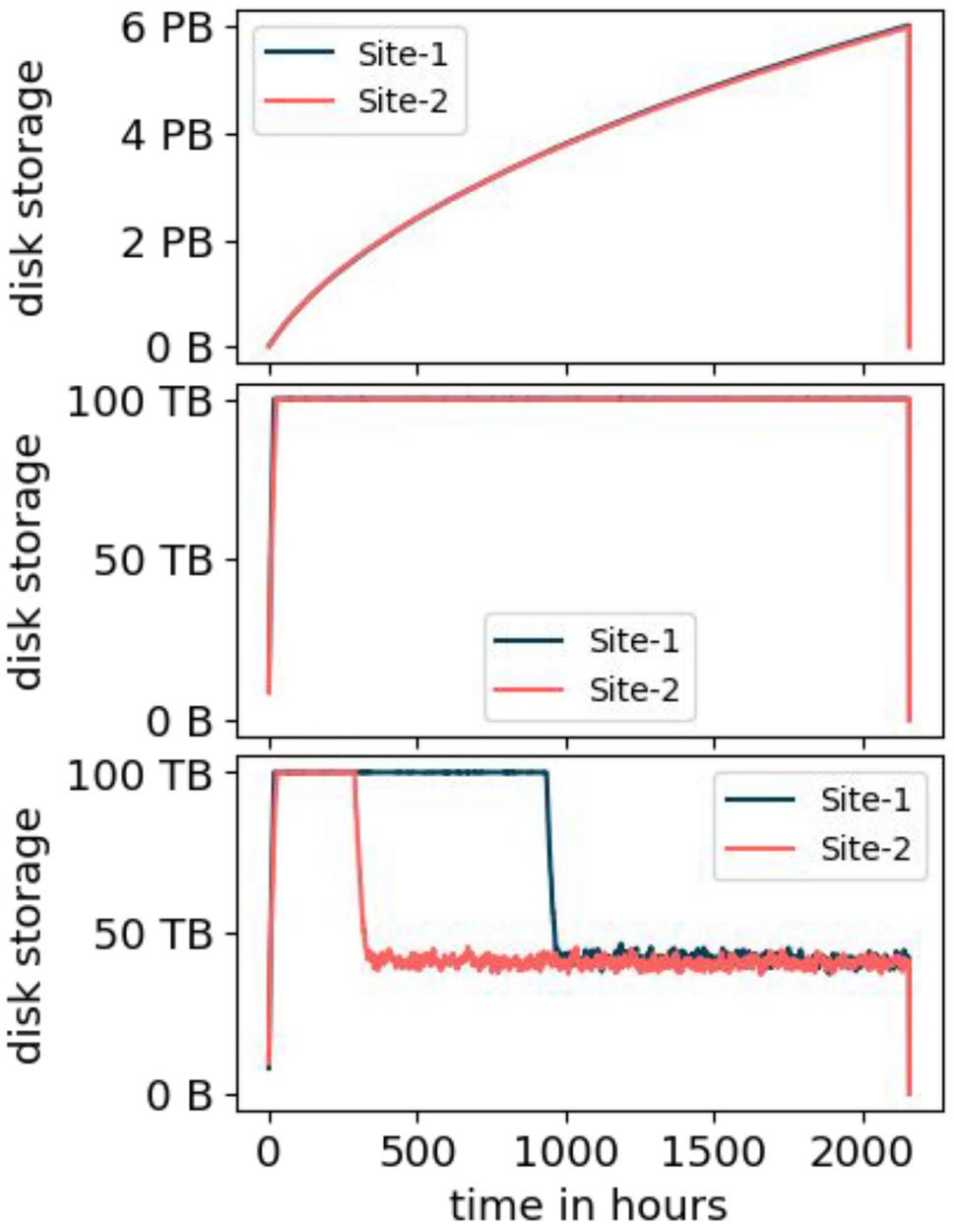
Increase of storage used at the disk storage element for *configuration I* (top), *II* (middle), and *III* (bottom). The increase of the storage used at Site-1 and Site-2 overlap because they are very similar for *configuration I* and *II*

Figure 6 shows the increase of the storage used over time for each configuration and each disk storage element. In *configuration I*, the disk storage element is unlimited. This results in a quick increase of storage used at the beginning. The more data is transferred to the disk storage, the more the increase flattens out.

The disk storage of *configuration II* is limited to 100 TB as reflected by the corresponding graph in Fig. 6. The storage used fluctuates slightly below 100 TB because of the continuous deletion of old and creation of new replicas.

At the beginning of the simulation, *configuration III* is equal to *configuration II* because all the data is at the tape storage element and is required to be transferred to the disk storage element prior to its processing. During this time, more jobs are submitted on average than can be processed because of the tape access latency and the limit of number of transfers. This results in a backlog of jobs waiting for their data to be transferred to the disk storage element. For *configuration III*, a sufficient amount of data is stored at GCS at some point. Afterwards, more data is transferred from GCS than from tape and thus, the tape access latency and limited number of transfers become less important. This results in quick processing of the jobs backlog because the rate of processed jobs exceeds the rate of newly submitted jobs. Moreover, the amount of required disk storage is reduced because fewer jobs are running at the same time. Site-2 requires less time to reach this point because the configured tape throughput is larger than at Site-1.

As mentioned before, additional tests with a random normally distributed tape access latency were made. The access latency was in the range between 0 and 90 minutes. The first runs were made with a mean value of 30 minutes and a standard deviation of 10 minutes. The number of finished jobs and the volume transferred did not change significantly for any configuration. For *configuration II*, an increased standard deviation slightly reduced the number of jobs because at some point the transfers with large access latency dominate and create a transfer queue backlog. The mean value of the random distribution has a noticeably stronger impact. Increasing the mean to 60 minutes ± 15 minutes reduced the number of finished jobs by $\approx 20$% for *configuration II*. The number of finished jobs for *configuration I* and *III* were reduced by 2% and 4%, respectively.

Approximately the same number of jobs are submitted for each configuration, but in the case of *configuration II* $\approx 15\%$ fewer jobs are finished. This leads to the conclusion that some jobs spent more time in early states of the job object state machine. Each job object stores different time points from its submission to its deletion. The *job waiting time* is the time span from the submission of a job until the job is queued. This includes the time the job must wait for disk storage, the time that the corresponding transfer spends in the queue, and the time to finish the transfer.

**Fig. 7:**
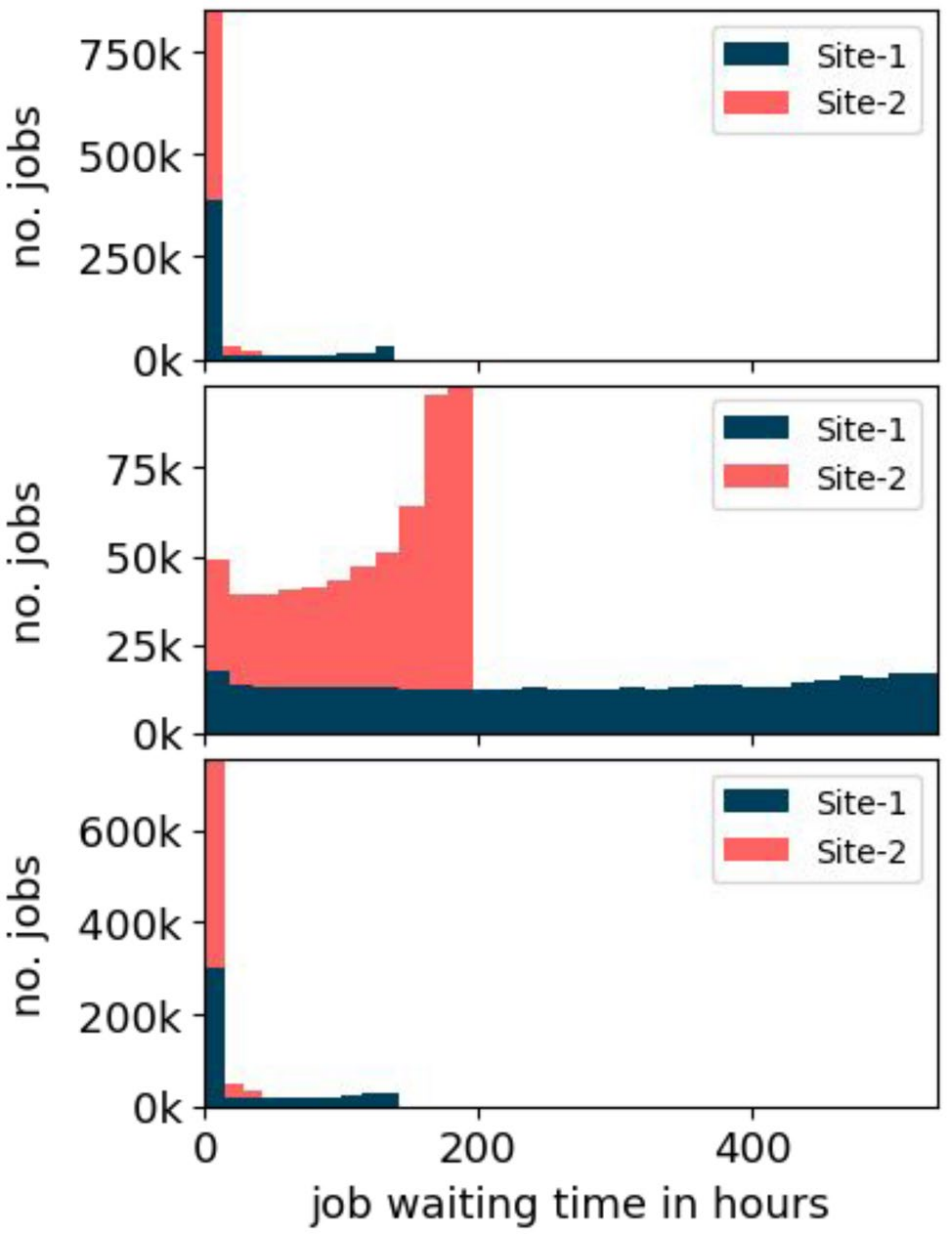
Job waiting time distribution of the HCDC model for *configuration I* (top), *II* (middle), and *III* (bottom). The number of bins in the top and bottom histogram was reduced from 30 to 10 to improve visibility of the first bin. The bins are stacked

Figure 7 shows histograms for the job waiting time for each configuration. The top histogram shows the data from *configuration I*. As expected, the vast majority of jobs have a small job waiting time because the disk storage is not limited and the data must be transferred only once from the tape storage element to the disk storage element. At the beginning of the simulation, the same job backlog builds up as explained earlier for *configuration III*. The transfers at the end of this backlog represent the outliers in the histogram with a job waiting time of $\approx 150$ hours.

The second histogram shows the data from *configuration II*. The histogram shows that more jobs have a larger job waiting time when the disk storage is limited. The job waiting time distribution is different between Site-1 and Site-2. The histogram shows that the distribution of job waiting time for Site-1 is $\approx 2.5$ times larger than for Site-2. As in *configuration III*, a backlog of waiting jobs develops in *configuration II*. In contrast to *configuration I* and *configuration III*, the job processing rate will not exceed the rate of newly submitted jobs because the disk storage is limited and GCS is not used. This means the data must be frequently transferred from tape. Thus, the job backlog will not be processed completely.

In *configuration II*, three types of jobs are responsible for the job waiting time close to 0 hours. (i) The very first jobs at the beginning of the simulation. (ii) Jobs that require data which already exists at the disk storage at the time of the job submission. (iii) Jobs that are already being transferred to the disk storage at the time of the job submission. Larger job waiting times indicate jobs that were inserted further towards the end of the backlog. In addition, larger file sizes, larger transfer times, and unpopular data potentially increase the job waiting time.

The third histogram shows the data from *configuration III*. The result is similar to the histogram of *configuration I*, but it contains more jobs with durations above 0 hours. This can be explained by the inclusion of the transfer duration into the waiting time. In this case, the transfer time from the GCS bucket to the disk storage element is added.

**Fig. 8:**
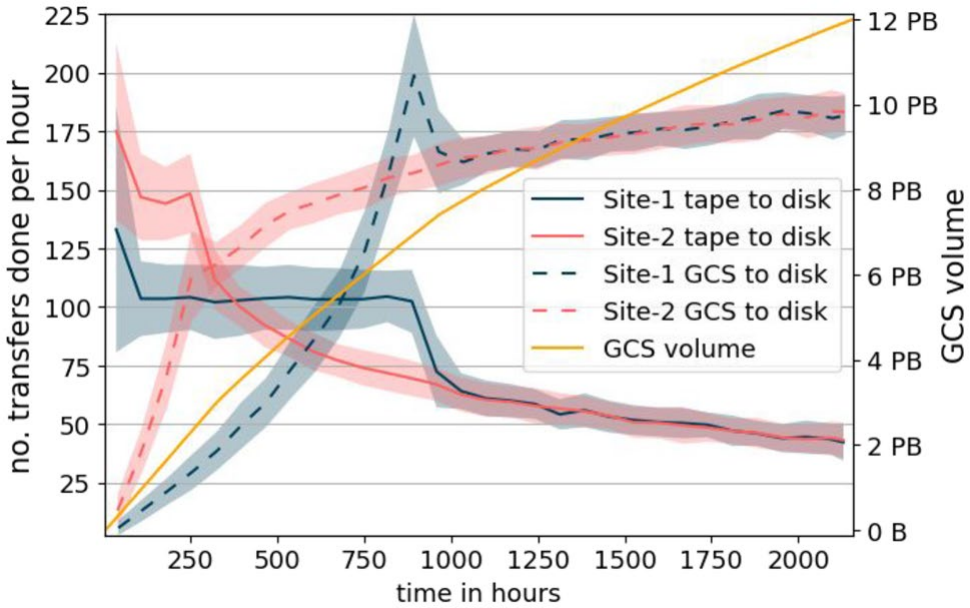
The solid blue and red line show the number of transfers from tape to disk per hour for each site. The dashed lines show the number of transfers from GCS to disk per hour for each site. The orange line shows the GCS volume used

Figure 8 illustrates the usage of GCS. This graphic is only available for *configuration III* because it is the only configuration using GCS. Data for the blue and red lines is aggregated in the form of count per hour. To reduce the fluctuations of these aggregated values, a mean filter was applied. The corresponding standard deviation is shown by the line contours.

The blue and red lines show the number of transfers for Site-1 and Site-2, respectively. The solid versions of these lines show the hourly number of transfers from tape to disk, while the dashed versions show the hourly number of transfers from GCS to disk. The orange line shows the volume of GCS used.

Since all the data is solely on tape storage at the beginning, the number of transfers from GCS to disk and the GCS volume used start at 0. The blue and red solid lines show that most data is transferred from tape to disk storage at the beginning.

All the data that is transferred from the tape to the disk storage is subsequently transferred from the disk storage to GCS. Furthermore, this implementation of the model does not delete the data at GCS. This means the orange line increases dependent on the solid lines. At some point, the most popular data is stored at GCS. This is when the dashed line exceeds the corresponding solid line. This means more data can be transferred from GCS to the disk storage than from the tape storage to the disk storage. The increase of the stored volume at GCS flattens as the most popular data is replicated to GCS.

The figure also reflects the full disk storage in the first half of the simulation. As explained earlier, there is an initial job backlog due to the tape access latency and limited number of transfers. Because of this, the disk storage limit is reached. The full disk storage leads to a steadily growing job backlog because new jobs must wait for disk storage space. Even new jobs whose data is already on GCS must wait for disk storage. As the amount of data at GCS increases, more and more of the newly submitted jobs use GCS as a data source. This allows processing the jobs faster than new jobs are submitted on average. The peak in the dashed lines indicates the time when the backlog has been processed.

**Table 7:** Mean and standard deviation of transferred volume between storage elements for *configuration I, II,* and *III* of 20 simulation runs

Cfg.	Site	Transfer	Volume (SE)
*I*	Site-1	Tape to disk	6.75 PB ± 0.28% (0.06%)
*I*	Site-2	Tape to disk	6.74 PB ± 0.29% (0.06%)
*II*	Site-1	Tape to disk	8.85 PB ± 0.07% (0.04%)
*II*	Site-2	Tape to disk	13.04 PB ± 0.20% (0.01%)
*III*	Site-1	Tape to disk	6.74 PB ± 0.30% (0.07%)
*III*	Site-2	Tape to disk	6.75 PB ± 0.19% (0.04%)
*III*	GCS	GCS to disk	24.99 PB ± 0.46% (0.10%)

Table 7 shows detailed numbers of the transfer statistics. The transferred volume of Site-1 and Site-2 for *configuration I* is almost the same. This is because at some point, the most popular data is available at the disk storage and does not need to be transferred again from the tape storage. Around 13.5 PB of data was transferred to the disk storage. Comparing this amount to the volume downloaded from disk to worker storage of 41.11 PB from Table 6 underlines the conclusion that data is reused.

The transferred volume for *configuration II* shows that much more data is required to be transferred from tape storage. This is because the data at the disk storage is deleted after processing and has to be re-transferred in case it is required again. The difference of the transferred volume between Site-1 and Site-2 makes clear that the tape-to-disk transfer performance is the bottleneck.

The transferred volume from tape to disk storage for *configuration III* shows similar numbers to *configuration I*. The volume of 6.75 PB seems to be the required storage for the most popular data. Once this data is available at the disk storage for *configuration I* or at the GCS for *configuration III*, the tape-to-disk transfer performance becomes less important.

The volume transferred from GCS to disk is split between both sites, which makes a mean of $\approx 1.6$ GB/s per site over 3 months. As mentioned before, the configured throughput for the network links is based on small-scale real-world tests. This configuration must be adjusted when increasing the scale of the simulation in order to achieve realistic results. The real-world tests of the VR Observatory [16] resulted in a similar throughput estimation. Using a simple regression over 4 data points, they calculated a bandwidth of $\approx 1.5$ GB/s without special performance tuning.

At the end of the simulation there is $\approx 12$ PB of data stored at GCS. $\approx 6.8$ PB have not been transferred out of GCS during the simulated time. The number of times a file was recalled from GCS is in the range from 0 to 45. The files that were recalled less than 25 times are responsible for more than 90% of the traffic from GCS to the site disk storage.

**Table 8:** Mean GCS costs for each month with the standard deviation and standard error for 20 simulation runs. Pricing information were taken in US Dollar (USD) from the GCP documentation at 2020-09-10

Month	Storage cost in USD (SE)	Network cost in USD (SE)
1	82k ± 0.10% (0.02%)	330k ± 0.39% (0.08%)
2	211k ± 0.16% (0.03%)	729k ± 0.31% (0.07%)
3	293k ± 0.23% (0.05%)	807k ± 0.25% (0.05%)

Table 8 lists the mean cost of the cloud resources per month used by the simulated model. The pricing information was taken from the GCP documentation on 2020-09-10. The storage cost increases from the first month on because at the beginning the data is solely on tape and is gradually transferred to the cloud storage. With an increasing amount of data at GCS, more data can be transferred from GCS to the disk storage. Thus, the network cost increases from month to month. The network cost is in general larger than the storage cost, since the price for network traffic is typically much higher than the storage price.

## Conclusion

This contribution describes the data carousel model and the Hot/Cold Storage model. Currently, both models are being developed and evaluated by the ATLAS collaboration. Specific workflows can benefit from a combination of these two models into the HCDC model. The HCDC model can be used in different variations, e.g., employing different storage types to implement the Hot/Cold Storage model part or applying different workflows to improve the impact of the data carousel model part.

To evaluate variations of the HCDC model and estimate certain metrics, a simulation was developed. The simulation was developed as a multi-purpose software framework. This framework can be used to simulate various kinds of models related to distributed computing and commercial cloud storage and network resources.

The simulation was executed on a virtual machine with 8 GB memory and 4 CPU cores clocked at 2.4 GHz. The validation model required less than a second in real time to simulate one day. The more complex HCDC model required less than two seconds in real time for one simulated day. The memory consumption mainly depends on the number of files and replicas. Both types require 68 bytes for each object instance. The memory consumption of the HCDC simulation starts at $\approx 480$ MB and peaks at $\approx 500$ MB during run time.

To validate the basic functionality of the simulation framework, an existing workflow with sufficient monitoring data was simulated and evaluated. The workflow used was the transfer of ATLAS derivation input data between sites.

Section 5.4 discussed the evaluation of the simulation of the HCDC model. The HCDC model implementation used GCS for cold storage and assumed a continuous production of derivation data. With a processed volume of 41 PB of input data in 3 months and a re-read rate of 25 times or less for 90% of the data, 12 PB of disk storage are required to be able to keep the data solely on tape storage without throttling the job throughput. Using the HCDC model, the on-premises disk storage limit can be further reduced to 100 TB by employing additional cloud storage without reducing the number of finished jobs. However, the GCS and network usage introduce additional costs. Whether these costs are worth the additional throughput has to be decided individually for each institution of a collaboration.

A simulation tool, such as the presented simulation, can assist by calculating the parameters for the decision whether to use cloud resources. Assuming the HCDC model, parameters can be considered as fixed or variable. Fixed parameters are dictated by the problem description and the existing resources, e.g., bandwidths, input volume to process, number of jobs to run, or the popularity of files. Variable parameters can be changed directly or change indirectly depending on other variable parameters. For example, the cost and the job throughput change depending on a potential GCS limit. The time limit to process all the data change depending on the job throughput. The required GCS limit depends on the available disk storage limit. Given a specific use case with well-defined limits of the variable parameters, the simulation can be used to estimate the optimal balance among the parameters.

A typical consideration is to compare the cost introduced by the cloud provider against the benefits of the additional resources. In Sect. 5.4, the benefits of additional resources were measured in form of number of jobs finished resulting from the input volume throughput. From these values, more specific metrics can be derived, e.g., job slot saturation or the volume of output data. For example, *configuration I* required $\approx 12$ PB more disk storage but *configuration II* finished $\approx 15$ % fewer jobs. *Configuration III* added more than 2 million USD of cost for three months.

There are two topics that should be prioritised in future work. First, implementing additional concepts to optimise the cost and performance of the model. Second, improving the parameters used and the underlying assumptions in order to achieve more accurate and realistic results. When these topics have been addressed, a specification could be defined for the variable parameters and the simulation can be used to search the optimal values. These values can be used for real-world tests to analyse the accuracy of the estimation of the simulation.

In terms of the first topic, there are two missing concepts that should be prioritised in future work. Currently, it is possible to limit the GCS, but there is no mechanism that deletes data on GCS. This is an essential concept to be able to define narrower limits on GCS and subsequently reduce storage cost. Different deletion strategies are possible, e.g., setting a storage capacity threshold. After surpassing this threshold, the data can be deleted from GCS based on the popularity.

The other concept that should be prioritised in future work is transfers between sites. Using WLCG resources, the data to process is typically distributed among various sites to benefit from different resources and optimally distribute the workload. The challenge of implementing this into the simulation is the creation of a realistic model that specifies the amount and the selection of data to transfer between sites.

When these two concepts are implemented, further adjustments to the HCDC model are possible. For example, the GCS egress cost is even more critical than the GCS storage cost. These could be reduced by improving the deletion at the disk storage element and making the caching strategy more intelligent. Moreover, the utilisation of the tape storage decreases as the GCS usage increases. Instead of always preferring GCS over the tape storage, both storage categories could be used optimally. One approach for this would be to store the largest files only on the tape storage and not transfer them to GCS. This is because the access to the tape storage becomes more performant for larger files. The tape storage usage would be improved and the egress cost of the cloud storage would be further reduced.

Another approach to potentially reduce egress cost is to utilise cloud computing resources and derive data directly inside the cloud. Although this approach adds cost for the computing resources, it reduces the egress cost because only the smaller derived data has to be transferred out of the cloud.

Further, future work could also investigate the HCDC model assuming the derivation production workflows being organised in campaigns. As explained in Sect. 3, in this case it is expected that the Hot/Cold Storage model part becomes less relevant but the data carousel model part can be used optimally.

The second topic that should be prioritised for future work is the improvement of the parameters used and models. The parameters used are very specifically fitted to the monitoring data. The parameters are realistic as long as the same monitoring data is used. However, changing one of the parameters might invalidate the other parameters. For this reason, the following improvements should be made. (i) As described in 5.3, the job submission model should be adjusted, e.g., based on a job slot limit per site. In the case of a job slot based model, it is also important to carefully consider other limitations of the storage systems, such as IOPS. (ii) The number of input files for each job must be generated based on a more realistic distribution. (iii) The network links should be configured with a shared bandwidth, which requires more detailed information about the real network topology. In addition, background traffic should be added to shared bandwidth links, assuming the links are not exclusively used for the simulated scenario. (iv) The throughput between GCS and the WLCG was based on rather small scale tests and need to be tested with larger transfer volumes or modelled differently. (v) The simulated HCDC model used a statically assigned popularity based on a fitted random function. This could be replaced by a dynamic assignment. A straightforward approach would be the least recently used concept, which is an established CPU caching technique. More advanced approaches could implement models of existing research about popularity based data replication [21, 22]. (vi) The tape access latency is a strong simplification of real tape systems. A more realistic model based on logs of a real tape system would be required. (vii) The effect of increasing the simulation time has to be investigated in future work. The simulation scales to much larger times, e.g., one year. However, this requires implementing the improvements of the parameters because the current parameters might not be valid for more than 3 months.

## Data Availability

The data that support the findings of this study are available from the ATLAS Collaboration, but restrictions apply to the availability of these data, which were used under licence for the current study, and so are not publicly available. Data are however available from the authors upon reasonable request and with permission of the ATLAS Collaboration. This manuscript has associated data in a data repository. [Authors’ comment: The used monitoring data were taken from the ATLAS monitoring system and are available upon request and with permission of the ATLAS Collaboration.]

## Footnotes

1 The distributed computing infrastructure and activity was not unduly impacted by the global COVID-19 pandemic.
